# Preparation and Permeation Properties of a pH-Responsive Polyacrylic Acid Coated Porous Alumina Membrane

**DOI:** 10.3390/membranes13010082

**Published:** 2023-01-09

**Authors:** Takafumi Sato, Kotomi Makino, Shingo Tamesue, Gakuto Ishiura, Naotsugu Itoh

**Affiliations:** 1 Department of Fundamental Engineering, Utsunomiya University, Yoto, Utsunomiya 7-1-2, Tochigi, Japan; 2 Division of Engineering and Agriculture, Utsunomiya University, Yoto, Utsunomiya 7-1-2, Tochigi, Japan

**Keywords:** pH-responsive membrane, polyacrylic acid, porous alumina support, membrane coating

## Abstract

A pH-responsive membrane is expected to be used for applications such as drug delivery, controlling chemical release, bioprocessing, and water treatment. Polyacrylic acid (PAA) is a pH-responsive polymer that swells at high pH. A tubular α-alumina porous support was coated with PAA by grafting to introduce appropriate functional groups, followed by polymerization with acrylic acid. The permeances of acetic acid, lactic acid, phenol, and caffeine were evaluated by circulating water inside the membrane, measuring the concentration of species that permeated into the water, and analyzing the results with the permeation model. The permeance of all species decreased with increasing pH, and that of phenol was the largest among these species. At high pH, the PAA carboxy group in the membrane dissociated into carboxy ions and protons, causing the swelling of PAA due to electrical repulsion between the negative charges of the PAA chain, which decreased the pore size of the membrane and suppressed permeation. Furthermore, the electrical repulsion between negatively charged species and the PAA membrane also suppressed the permeation. The results of this study demonstrated that the PAA-coated α-alumina porous support functioned as a pH-responsive membrane.

## 1. Introduction

In recent years, pH-responsive permeation systems have attracted attention for applications including drug delivery, controlling chemical release, biological and chemical separation, and water purification, and they are also an important technology for developing high-performance separation systems. These pH-responsive membranes are expected to be a major method of enabling easy separation according to the pH of the environment and they are generally prepared from pH-responsive polymers [1]. For example, membranes have been fabricated from pH-responsive polymers including poly(methacrylic acid-co-ethylene glycol dimethacrylate) [2], polyacrylic acid (PAA) or polymers containing PAA [3,4,5,6,7,8,9,10,11,12,13,14,15,16], calcium ion crosslinked sodium alginate on polyvinylidene fluoride [17], dimethylamino ethyl methacrylate-4-vinylpyridine [18], polypyrrole-polysulfone [19], poly(*N*-isopropylacrylamide-co-methylacrylic acid) [20], poly(vinylidene-fluoride-co-hexafluoro-propylene) [21], poly(*N*,*N*′-dimethylamino-2-ethyl methacrylate) [22], poly(carboxybetaine methacrylamide-co-*N*-(hydroxymethyl) acrylamide [23], and poly(4-vinylpyridine)-co-acrylonitrile [24].

PAA is a widely used polymer because its structure is simple and it swells at high pH. Structures containing PAA as a gate membrane include a PAA-coated porous alumina support [3], PAA-coated porous polymers [4,5,6,7], a PAA-coated nanosheet [8], and a PAA-grafted carbon nanotube-intercalated membrane [9]. Mixed polymers consisting of PAA combined with other polymers are also used as pH-responsive membranes, and phase separation sometimes occurs to form a sponge-like structure. Examples include polyvinylidene fluoride-PAA [10], polyvinyl pyrrolidine-PAA [11], polystyrene-PAA [12], PAA-poly-*N*-isopropylacrylamide [13], PAA-polyvinylidene fluoride [14], and polyvinyl alcohol-PAA [15]. A membrane containing PAA-coated ZnO particles exhibited pH responsiveness [16].

Gate membranes with a stable inorganic material such as α-alumina as a support have shown excellent durability and chemical stability. Industrial uses require a support with high mechanical strength and a simple coating method; however, the inorganic support for reported PAA gate membranes is a thin alumina disk that is fragile, and specific coating methods, such as plasma coating, have been used. Furthermore, the pH responsiveness for the PAA membrane has been evaluated by the permeation of a solvent [3,5,6,7,8,9,10,11,12,13,14,15], caffeine [4,5], sugar [6,7], a pigment [8], phosphate [9], copper [10], dextran [11,12,15], bovine serum albumin [11], vitamin B12 [15], tryptophan [15], and urea [15]. To the best of the authors’ knowledge, there are no studies that evaluate the permeance of various species for the same membrane. We think that the preparation of PAA-coated membranes to the alumina porous tube with a general method that realizes industrial utilization and the comparing of the differences in permeance of chemical species for the same membrane are meaningful topics.

In this study, a tubular porous support made from stable α-alumina was used that would be suitable for industrial applications. The membrane that an α-alumina porous support coated with PAA was prepared by general organic synthesis, and the effect of pH on permeance was elucidated for several chemical species to evaluate the pH-responsive properties of the membrane.

## 2. Materials and Methods

### 2.1. Materials

The porous α-alumina porous support (o.d. of 1.9–2.3 mm, average pore diameter of about 150 nm) was supplied by AIST (Japan). Tetraethyl orthosilicate (TEOS; >95.0%), HCl (35%), acryloyl chloride (>98.0%), acrylic acid (>98.0%), ammonium peroxodisulfate (APS; >98.0%), toluene (>95%), tetrahydrofuran (THF; >99.5%), acetic acid (>99.7%), L-lactic acid (85.0%–92.0%), phenol (>99.0%), 0.2 mol/L HCl, and 0.2 mol/L sodium hydroxide aqueous solution were purchased from FUJIFILM Wako Pure Chemical Corp., Osaka, Japan. 4-(4,6-Dimethoxy-1,3,5-triazin-2-yl)-4-methylmorpholinium chloride (>95.0%), ethanol (>99.5%), methanol (>99.8%), sodium tetraborate decahydrate (99.5–101.0%), disodium hydrogen phosphate dodecahydrate (>99.0%), and sodium dihydrogen phosphate dihydrate (99.0–102.0%) were purchased from Kanto Chemical Co., Ltd., Tokyo, Japan. 3-Aminopropyltrimethoxysilane (APTMS; >96.0%) was purchased from Tokyo Chemical Industry Co., Ltd., Tokyo, Japan. Sodium acetate (>98.0%) was purchased from Nacalai Tesque Inc., Kyoto, Japan. All chemicals were used without purification. Water was obtained from a water purification system (WG-204, Yamato Co., Tokyo, Japan).

### 2.2. Synthesis of PAA-Coated Alumina Membrane

**Figure 1:**
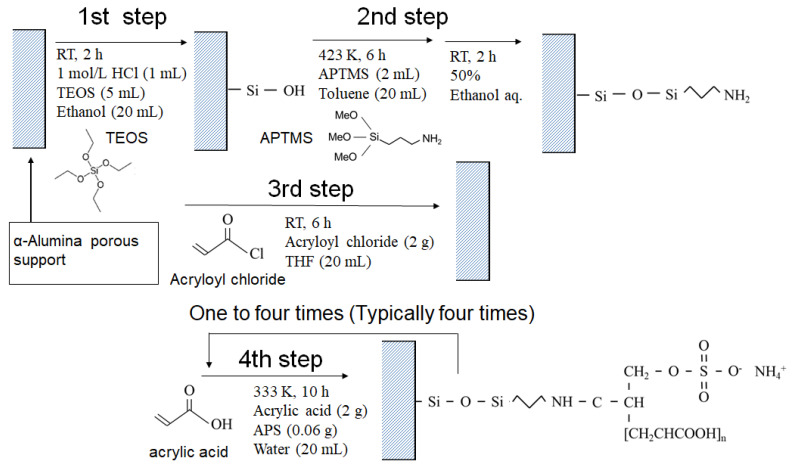
Synthetic route for the PAA-coated porous membrane.

Figure 1 shows the synthetic route for the PAA-coated alumina membrane. There were four steps in the membrane synthesis, and it included a modified reported method for PAA introduction to alumina particles [25]. In the first step, TEOS (5 mL) was added to methanol (20 mL), and then 1.0 mol/L HCl (1 mL) was added to this solution and stirred for 6 h. The alumina support was immersed in this solution and stirred for 2 h. The support was washed with methanol and dried at 333 K overnight. In the second step, APTMS (2 mL) was dissolved in toluene (20 mL). The support obtained in the first step was immersed in the toluene solution and the solution was refluxed at 423 K for 6 h under stirring. The support was washed with ethanol, and then was placed in ethanol–water (1:1 *v*/*v*) for 2 h. In the third step, acryloyl chloride (2.0 g) was dissolved in THF (20 mL) and the porous support obtained in the second step was immersed in the solution with stirring at room temperature for 6 h. After washing, the support was dried in a desiccator under reduced pressure. In the fourth step, acrylic acid (2.0 g) was diluted with water (20 mL) and APS (0.06 g) was added. The support obtained from the third step was immersed in this solution at 333 K for 10 h under stirring to allow the polymerization to proceed. After the reaction, the support was washed with water and dried under reduced pressure for 2 h. The polymerization was typically repeated four times and membranes were also synthesized with one to three polymerizations for comparison.

### 2.3. Analysis of PAA-Coated Alumina Membrane

A sample was cut from the membrane obtained in each step. A piece of each sample was coated with Pt by a Pt-Pd ion coater (E-1030, Hitachi Ltd., Tokyo, Japan), observed by scanning electron microscopy (SEM; JSM-5610LV, JEOL Ltd., Tokyo, Japan), and analyzed by thermogravimetric analysis (TGA; Thermo Plus Evo II, Rigaku Corp., Tokyo, Japan) under a nitrogen flow of 150 mL/min up to 1173 K. The membrane was also crushed, and the obtained powder was sandwiched with KBr plates. The components of the powder were analyzed by FT–IR (FT/IR-4100, JASCO Corp., Tokyo, Japan).

Titration experiments for the PAA-coated alumina membrane and uncoated support were conducted as follows. The membrane or support was submerged in aqueous solution containing 2.5 × 10^−4^ mol/L HCl and 2.5 × 10^−2^ mol/L NaCl (80 mL). An aqueous base solution containing 2.0 × 10^−3^ mol/L NaOH and 2.5 × 10^−2^ mol/L NaCl was then added dropwise to the acidic solution. NaCl was used to keep the ion strength constant. The pH of the solution was monitored with a pH meter (D-74, Horiba Ltd., Kyoto, Japan). The titration without the membrane and support was also conducted.

### 2.4. Permeation Test

**Figure 2:**
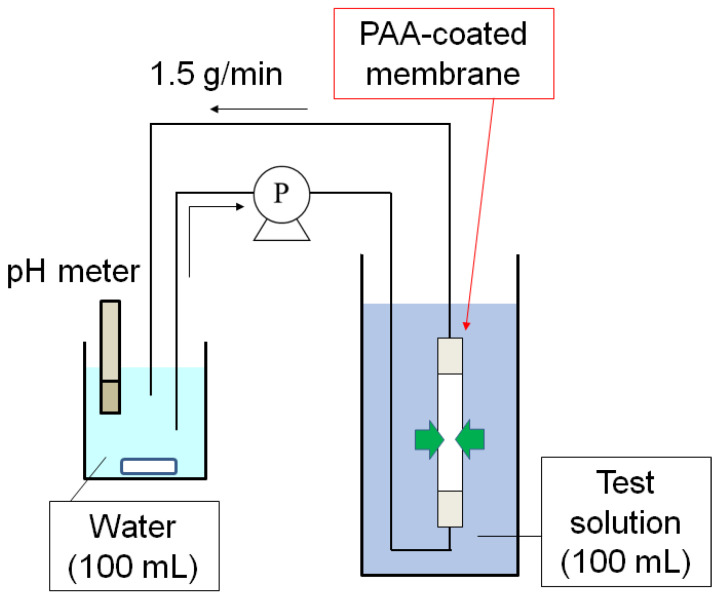
Permeation test system with circulation.

The permeations of species in single and mixed aqueous solutions of acetic acid, lactic acid, phenol, and caffeine through the PAA-coated alumina membrane were evaluated with a circulation system (Figure 2). The PAA-coated alumina membrane was covered with Teflon tape at both ends so that the exposed length was 50 mm, and then the membrane was submerged in test solution (100 mL) in a beaker. A second beaker was filled with water (100 mL) under stirring and the water was circulated through the inside of the membrane tube in the first beaker with a pump (TP-10SA, As One Corp., Osaka, Japan) at 1.5 g/min. The pH of the solution was typically measured with a pH meter (D-74, Horiba Ltd., Kyoto, Japan) in the second beaker. A few milliliters of the sample were taken at each predetermined time and analyzed with an HPLC-UV system (Prominence, Shimadzu Corp., Kyoto, Japan) containing an Inertsil ODS-4 column (GL Science Inc., Tokyo, Japan). Acetic acid and lactic acid were detected at 212 nm with a mobile phase of 10 mM aqueous H_3_PO_4_. Phenol and caffeine were detected at 254 nm with a mobile phase of 30 or 50 vol% aqueous acetonitrile.

The pH of 0.1 mol/L acetic acid was adjusted with HCl (pH 1.2), without additive (pH 2.8), with NaOH (pH 4.6 and 6.9), and with a mixture of disodium hydrogen phosphate dodecahydrate and sodium dihydrogen phosphate dihydrate (pH 6.2). Sodium acetate was used as an acetic acid solution at pH 8.0. The pH of 0.1 mol/L lactic acid was adjusted with HCl (pH 1.5), without additive (pH 2.3), with NaOH (pH 3.8), and with a mixture of disodium hydrogen phosphate dodecahydrate and sodium dihydrogen phosphate dihydrate (pH 6.4). The pH of 0.1 mol/L phenol was adjusted with HCl (pH 1.1), without additive (pH 5.9), with sodium tetraborate decahydrate (pH 9.4), and with NaOH (pH 11.4). The pH of 0.02 mol/L caffeine was adjusted with HCl (pH1.6), without additive (pH 6.4), and with sodium tetraborate decahydrate (pH 9.4). The pH of the 0.1 mol/L acetic acid + 0.1 mol/L phenol + 0.02 mol/L caffeine aqueous solution was adjusted with HCl (pH 1.1), without additive (pH 2.9), and with sodium tetraborate decahydrate (pH 8.7).

## 3. Results

### 3.1. Characterization of PAA-Coated Porous Alumina Membrane

SEM images of the cross section of the uncoated alumina support and the membranes obtained after the first, second, third, and fourth steps with four polymerizations are shown in Figure S1 in Supplementary Materials. In all cases, the alumina structure was observed and there were few changes from the first to the third step, which is probably because little inorganic and organic material was introduced into the alumina support. However, after the fourth step, there were small filamentous structures in the membrane that were probably derived from PAA introduced into the inner support. Further, other SEM images of the porous support surface for raw support, that after first step, that after second step, that after third step, that after fourth step with four polymerizations were almost the same. From these results, the PAA layer at the surface of support was not confirmed. These facts indicate that PAA was mainly in the inner side of support.

**Figure 3:**
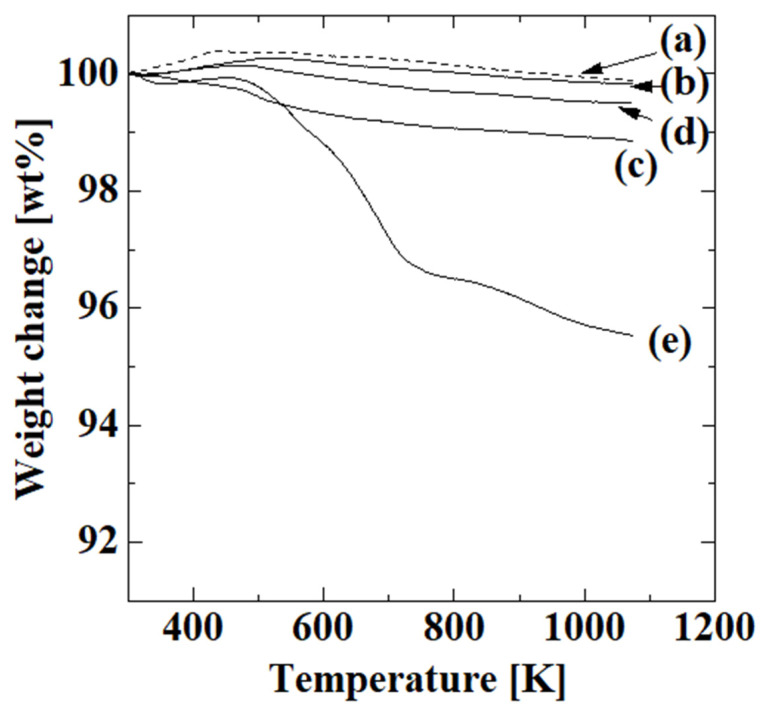
TGA results for the uncoated alumina support (**a**) and membranes obtained after first (**b**), second (**c**), third (**d**), and fourth steps with four polymerizations (**e**).

Figure 3 shows the TGA results for the uncoated alumina support and membranes obtained after the first, second, third, and fourth steps with four polymerizations. The thermogravimetric reduction curves of the first, second, and third steps indicate that there was no major weight loss, and the weight from the introduction of the -OH groups, -NH_2_ groups, and C=C double bonds was small. The weight of the membrane obtained after the second step was slightly lower than those of the uncoated alumina support and the membranes obtained after the first and third steps, which is probably because the ratio of the surface and inner side in each sample was different due to difficulties in the sample preparation. These individual differences among samples probably affected the thermogravimetric reduction curve. In contrast, the membrane obtained after the fourth step showed substantial weight loss due to dehydration of the absorbed water at 323–428 K [25], decomposition of the PAA carboxy group at 423–713 K [25,26,27], and decomposition of the PAA chain itself at 713–1073 K [25,27]. The corresponding weight losses were 0.2, 3.0, and 1.3 wt%, respectively, which indicated that PAA was introduced after the fourth step. The weight ratio of PAA in the sample was calculated from the 3 wt% weight loss derived from decarboxylation of PAA as a reasonable value of 5.1 wt%, which showed that acrylic acid polymerized on the alumina support.

**Figure 4:**
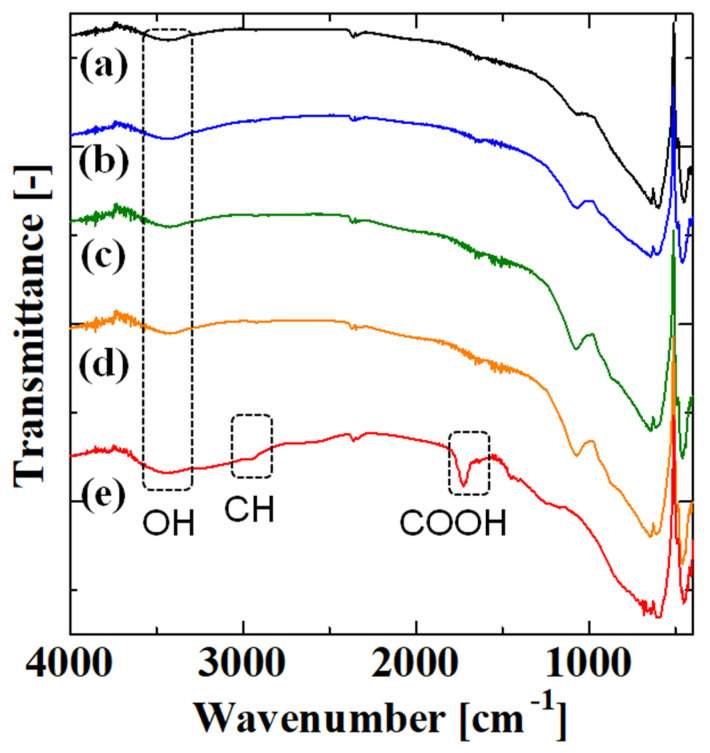
FT–IR spectra of the (**a**) uncoated alumina support and membranes obtained after the (**b**) first, (**c**) second, (**d**) third, and (**e**) fourth steps with four polymerizations.

Figure 4 shows the FT-IR spectra of the uncoated alumina support and membranes obtained after the first, second, third, and fourth steps with four polymerizations. We analyzed crushed power because PAA was mainly in the inner side of the porous support. The samples mainly consisted of alumina from the support, and the amount of organic materials such as PAA was small; thus, the intensity of peaks derived from PAA was small. In all spectra, there was a broad peak around 3400 cm^−1^ corresponding to OH stretching [3,25]. The spectra of the uncoated alumina support and the membranes after the first, second, and third steps were similar, which is probably because the amount of material introduced was small. However, in the spectrum for the membrane obtained after the fourth step, a peak appeared around 2965 cm^−1^. In previous work, the peaks at 2926 [8], 2918 [16], 2933 [25], and 2941 cm^−1^ [26] were attributed to C-H bonds. Thus, the peak around 2965 cm^−1^ was from C-H bonds in PAA. Furthermore, there was a large peak at 1725 cm^−1^. The peaks 1715 [3], 1720 [5,7], 1700 [8], 1710 [10,28], 1723 [16], 1739 [25], 1705 [26], and 1725 cm^−1^ [29] were attributed to the carboxy group of PAA. Therefore, the peak around 1725 cm^−1^ was probably due to the carboxy group of PAA. These results confirmed that PAA was introduced to the membrane after the fourth step with four polymerizations.

**Figure 5:**
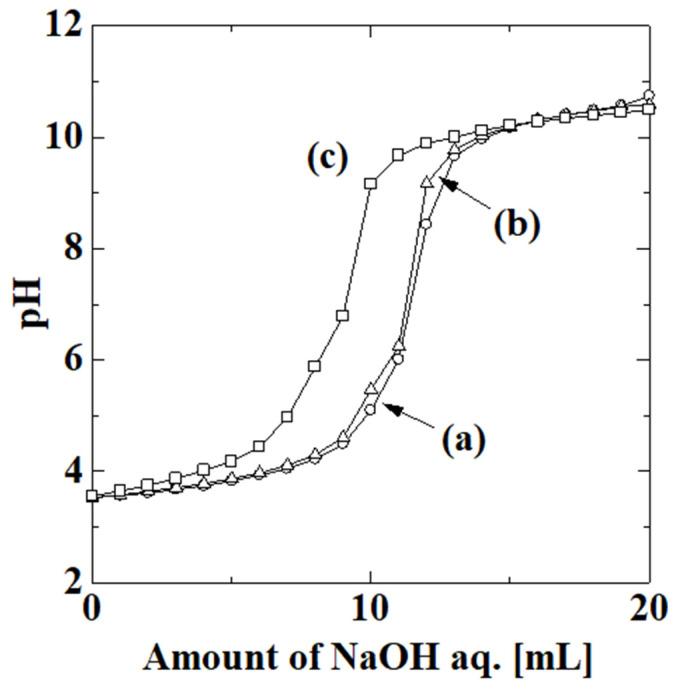
Titration curves of for (**a**, ○) membrane-free system, (**b**, Δ) uncoated alumina support, and (**c**, □) PAA-coated alumina membrane obtained after the fourth step with four polymerizations.

The surface of the PAA-coated alumina membrane contained hydroxy groups derived from the TEOS treatment and the PAA carboxy groups. The electron density of materials can be evaluated by titration [30], and thus the electron density of the membrane related to the surface properties during preparation was evaluated. Figure 5 shows the titration curves for (a) the membrane-free system, (b) the uncoated alumina support, and (c) the PAA-coated alumina membrane obtained after the fourth step with four polymerizations. The initial and final pH values of the membrane-free system, uncoated alumina support, and PAA-coated alumina membrane were 3.53 and 10.74, 3.53 and 10.58, and 3.56 and 10.49, respectively. The initial pH values were similar, as were the final pH values, regardless of the conditions. However, the shapes of the titration curves were different. In the absence of the support and membrane, the pH gradually increased with the amount of aqueous NaOH solution, increased sharply above 10 mL of NaOH, and then became almost constant. This is a typical trend for the titration of a strong acid with a strong base. In the presence of the uncoated alumina support, the amount of NaOH at which the sharp increase in pH occurred around 10 mL of NaOH was slightly lower than that in the absence of the membrane. The support did not affect the titration kinetics greatly, because of the stable properties of α-alumina. In the presence of the PAA-coated alumina membrane, the pH started to increase at less than 5 mL of NaOH, a sharp increase was observed below 10 mL of NaOH, followed by a gradual increase, and the pH became constant at more than 10 mL of NaOH. The support surface was treated with TEOS before PAA was introduced, so there were hydroxy groups derived from TEOS on the membrane and the hydroxy group quenched the HCl protons, thereby decreasing the amount of additional NaOH required to increase the pH. The effect of the surface hydroxy groups was probably larger than that of the carboxy groups on the membrane.

The electron densities of the alumina support and PAA-coated alumina membrane were calculated by the method in ref. [30] as 1.45 × 10^−6^ and 1.51 × 10^−5^ mol/g, respectively. The electron density of the PAA-coated alumina membrane was larger than that of the alumina support, which indicated that the properties of the PAA-coated alumina membrane were different from that of the alumina support. The contributions of the hydroxy group and PAA were unclear and should be examined in future work.

### 3.2. Evaluation of Permeation for PAA-Coated Alumina Membrane

**Figure 6:**
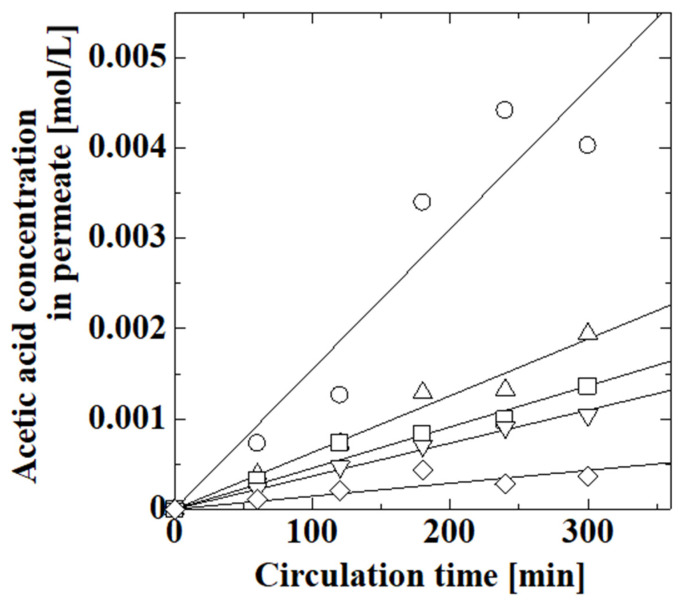
Effect of polymerization time on the permeation of acetic acid through PAA-coated alumina membrane for an acetic acid feed concentration of 0.1 mol/L: ○, uncoated alumina support; Δ, PAA-coated alumina membrane with one polymerization; □, PAA-coated alumina membrane with two polymerizations; ∇, PAA-coated alumina membrane with three polymerizations; and ◊, PAA-coated alumina membrane with four polymerizations.

The effect of the amount of polymer on membranes was evaluated by using the PAA-coated alumina membrane obtained with different numbers of polymerization reactions. We synthesized PAA-coated alumina membranes with one to four polymerizations in the fourth step of the synthesis, as described in Section 2.2. Figure 6 shows the effect of polymerization time on the permeation of acetic acid through the PAA-coated alumina membrane. The acetic acid concentration clearly decreased after the polymerization, which meant that the polymer synthesized on the support suppressed the permeation of acetic acid through the membrane. The acetic acid concentration decreased as the number of polymerization reactions increased. A new polymer layer was probably created in each polymerization step, and the membrane obtained after four polymerizations was used. The initial and final pH values of the feed solution for the membranes obtained by one, two, three, and four polymerizations were 2.93 and 2.95, 2.89 and 2.91, 2.91 and 2.92, and 2.84 and 2.85, respectively. The pH of the feed solution was almost constant during the experiment, and no change in liquid level was observed during the experiments, indicating that no one-way movement of water occurred between the feed and permeate.

We evaluated the effect of pH on the permeation of acetic acid, lactic acid, phenol, and caffeine through the PAA-coated alumina membrane. Table 1 summarizes the properties of the compounds used for the permeation test. Acetic acid is a basic carboxylic acid, lactic acid contains a hydroxy group in addition to an acetic acid carboxy group, phenol is a basic aromatic compound, and caffeine is a compound with a larger molecular weight.

**Figure 7:**
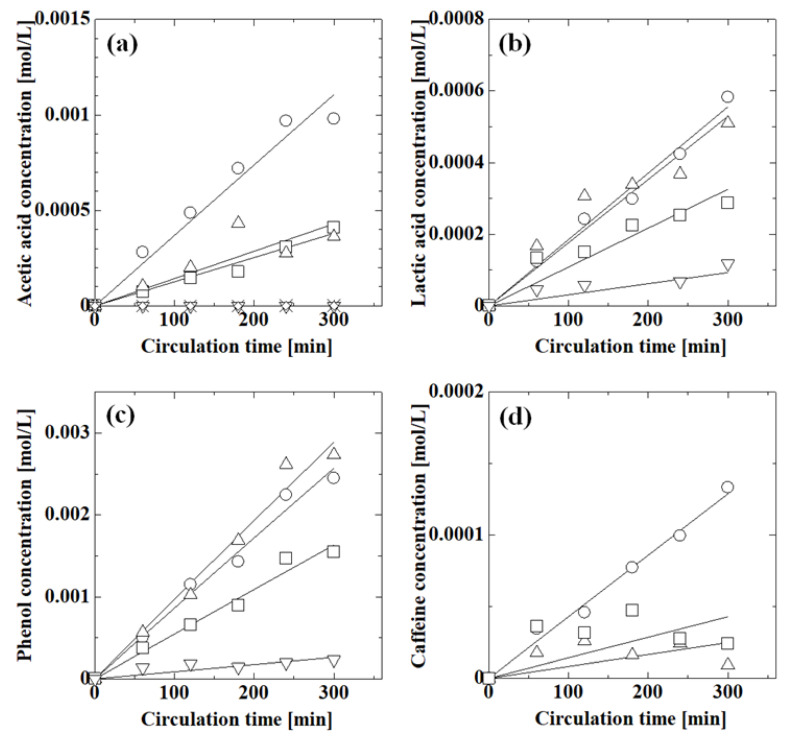
Effect of pH on the permeation of acetic acid, lactic acid, phenol, and caffeine through the PAA-coated alumina membrane obtained with four polymerizations: (**a**) 0.1 mol/L feed concentration of acetic acid at pH 1.2 (○), 2.8 (Δ), 4.6 (□), 6.2 (∇), 6.9 (◊), and 8.0 (×); (**b**) 0.1 mol/L feed concentration of lactic acid at pH 1.6 (○), 2.3 (Δ), 3.8 (□), and 6.4 (∇); (**c**) 0.1 mol/L feed concentration of phenol at pH 1.1 (○), 5.9 (Δ), 9.4 (□), and 11.4 (∇); (**d**) 0.02 mol/L feed concentration of caffeine at pH 1.6 (○), 6.4 (Δ), and 9.4 (□). Symbols: experimental values; lines: calculated values.

Figure 7 shows the effect of pH on the permeation of acetic acid, lactic acid, phenol, and caffeine. The concentrations of species increased with circulation time as the species permeated through the membrane. The acetic acid concentration decreased with increasing pH and was almost zero above pH 6.2; thus, pH-responsive permeation occurred across the PAA-coated alumina membrane. In the high-pH region, the PAA carboxy groups dissociated into carboxylate ions and protons, and electrostatic repulsion between the PAA carboxylate ions caused swelling of the PAA chain, which reduced the membrane pore size, thereby suppressing permeation at high pH. The lactic acid concentration decreased with increasing pH, similar to acetic acid. The actual lactic acid concentration was higher than that of acetic acid and the concentration increased with circulation time even at pH 6.4, which was probably because the interaction between lactic acid and the membrane was different from that of acetic acid. The phenol concentration at pH 1.5 was similar to that at pH 5.9; thus, the permeation in the low-pH region did not change much. However, the phenol concentration decreased with increasing pH above pH 5.9, indicating that the permeation of phenol was suppressed in the high-pH region. The phenol concentration was higher than those of acetic acid and lactic acid, although the molecular size of phenol was larger than those of acetic acid and lactic acid. The initial caffeine concentration was 0.02 mol/L because it was difficult to adjust the pH at 0.1 mol/L. The caffeine concentration at pH 1.6 was larger than those at pH 6.4 and 9.4. The concentration at pH 9.4 may have been larger than that at pH 6.4 due to experimental error because of the low caffeine concentration. Caffeine permeation was suppressed in the high-pH region, similar to the other species.

**Figure 8:**
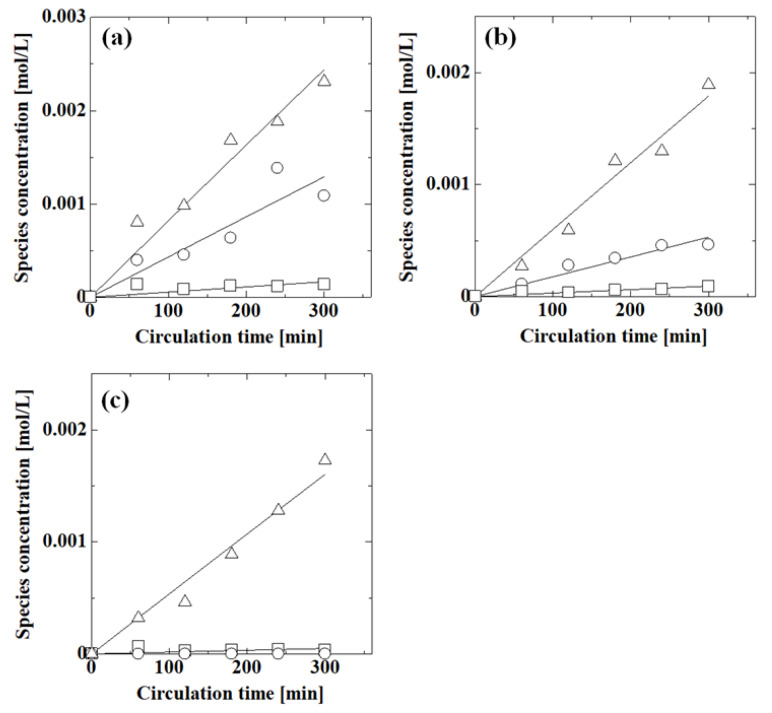
Effect of pH on the permeation of acetic acid, phenol, and caffeine in these mixtures at (**a**) pH 1.1, (**b**) pH 2.9, and (**c**) pH 8.7. Symbols: experimental values; lines: calculated values; ○: acetic acid; Δ: phenol; □: caffeine. Feed concentrations were 0.1 mol/L for acetic acid and phenol and 0.02 mL/L for caffeine.

Next, we evaluated the effect of pH on the permeation of each compound in mixtures of acetic acid, phenol, and caffeine. Figure 8 shows the effect of pH on the permeation of acetic acid, phenol, and caffeine in these mixtures. For each compound, the concentration decreased with increasing pH. The pH responsiveness of the membrane was also observed for mixtures. The phenol concentration was the highest of the three species. The acetic acid concentration was larger than that of caffeine at pH 1.1 and 2.9, although at pH 8.7, the acetic acid and caffeine concentrations were low. Phenol permeated the membrane more easily than acetic acid and caffeine.

The permeances of the species in all cases were calculated from the correlation of experimental data and the following typical permeation model.

(1) $$Q=PS\left(C_{h}-C\left(t\right)\right)$$

(2) $$\frac{dC\left(t\right)}{dt}=\frac{PSC_{h}}{V}-\frac{PS}{V}C\left(t\right)$$

(3) C(0)=0

Here, *Q* [mol/s] is the permeation rate of the species, *V* [m^3^] is the volume of circulated water, *C_h_* [mol/m^3^] is the initial concentration of the permeate species, *C*(*t*) [mol/m^3^] is the concentration of each species in the circulated aqueous solution, *S* [m^2^] is the surface area of the membrane, *P* [m/s] is the permeance, and *t* [s] is circulation time.

In this model, the permeation rate is proportional to the difference in concentration inside and outside the membrane (Equation (1)). The magnitude of the change in the concentration of the permeate species in the circulated aqueous solution changes with circulation time (Equation (2)), and the initial species concentration in the circulated aqueous solution is zero (Equation (3)). The initial concentration of permeate species was defined as constant, because this value was almost constant in the verification experiments. The permeance of each species was assumed to be constant.

For each set of conditions, the experimental concentration of each species with circulation time was correlated with the permeation model where the fitting parameter was the permeance of each species. The concentration calculated from the calculated permeance is shown by the lines in Figure 7 and Figure 8. The lines matched the experimental concentrations well, indicating that the permeation model was valid for evaluating the permeance of the system.

**Figure 9:**
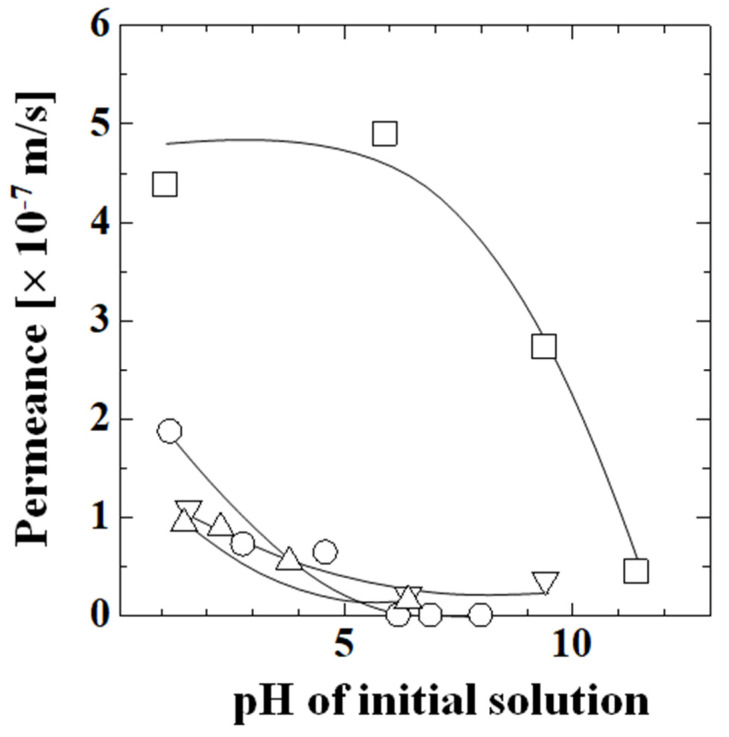
Permeance of each component in a single solution as a function of pH. Symbols: experimental values; lines: calculated values; ○: acetic acid; Δ: lactic acid; □: phenol; and ∇: caffeine.

Figure 9 shows the permeances of acetic acid, lactic acid, phenol, and caffeine in a single solution. The permeance of phenol was substantially larger than those of acetic acid, lactic acid, and caffeine. The permeance did not change with increasing pH below pH 5.9, sharply decreased above pH 6.0, and reached the same level as the other species at pH 11.4. The permeances of acetic acid, lactic acid, and caffeine were similar and decreased with increasing pH, and the decrease in permeance mainly occurred in the low-pH region below about pH 6.0. The permeance of acetic acid was zero above pH 6.2. The permeance of lactic acid was lower than that of acetic acid and it was not zero at pH 6.4, unlike acetic acid. The permeance of caffeine was similar to that of lactic acid and the magnitude of the decrease in permeance with increasing pH was small. The permeance changed by a factor of 0.19 from pH 1.6 to pH 6.4. The diffusion coefficient of caffeine changed by a factor of up to 0.28 times from pH 3 to pH 7 [5], which was a similar ratio to the change in permeation in this study.

Table 2 summarizes the permeance of acetic acid, phenol, and caffeine in a mixture in an aqueous solution. In all species, the permeance decreased with increasing pH. The permeation was suppressed at high pH in the mixture, as it was in the single aqueous solutions. The permeances of acetic acid in the mixture were similar to those obtained in the single aqueous solution (1.87 × 10^−7^ m/s at pH 1.2; 7.23 × 10^−8^ m/s at pH 2.8; 0 m/s above pH 6.2), as were those of phenol (single solution: 4.38 × 10^−7^ m/s at pH 1.1 and 2.73 × 10^−7^ m/s at pH 9.4) and caffeine (single solution: 1.09 × 10^−7^ m/s at pH 1.6 and 3.66 × 10^−8^ m/s at pH 9.4). The similarity of the permeances in the mixture to those in the corresponding single aqueous solution demonstrated that the interactions among the species did not affect the permeation in the concentration range examined.

## 4. Discussion

**Figure 10:**
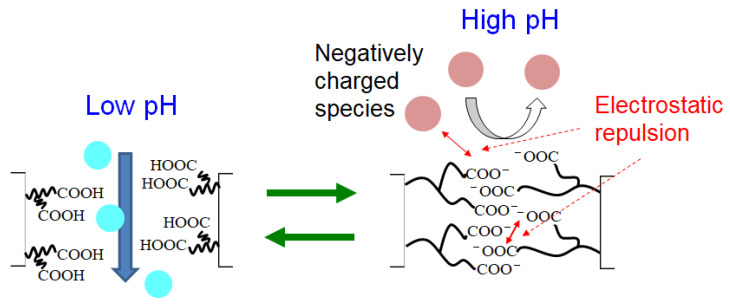
Proposed mechanism of pH dependence on permeation for the PAA-coated alumina membrane.

In this section, we discuss the permeation mechanism through the PAA-coated alumina membrane and we propose a mechanism for the permeation pH dependence (Figure 10). At low pH, the PAA carboxy groups in the membrane did not dissociate and the pore diameter of the membrane was large, allowing easy permeation by all species. As the pH increased, the PAA carboxy group dissociated to form protons and carboxy ions. The electrostatic repulsion among negatively charged carboxy ions caused the PAA polymer chains to swell, decreasing the membrane pore size with increasing pH. The membrane surface became negatively charged with increasing pH because of the PAA carboxy ions, and thus negatively charged species in solution were repelled from the membrane surface. The evaluation of pore size is interesting for considering permeation kinetics and will be a future challenge.

Next, we summarize the properties of species used for the permeation test. The molecular weight and pKa of species used for the permeation test are given in Table 1. The magnitude of the pKa was in the order of caffeine > phenol > acetic acid > lactic acid, and protons dissociate readily to form negative ions at low pKa. The molecular weight, which is a measure of the size of molecules, is in the order of caffeine > phenol > lactic acid > acetic acid. For molecules with the same negative charge, the smaller the molecular size, the higher the electrical density of the molecule is. The magnitude of the negative electrical density of the ions is in the order of acetic acid > lactic acid > phenol > caffeine, and the effect of the electrical repulsion between the molecule and membrane was probably also in this order. For example, the permeation rate of tryptophan decreases with increasing pH due to its ionization, and the permeation rate of vitamin B_12_ is almost constant regardless of pH due to low ionicity [15]. Furthermore, the magnitudes of the permeation of the molecule through the membrane generally obeyed the molecular size, and the permeance was acetic acid > lactic acid > phenol > caffeine. The overall permeation rate was probably determined by these factors.

The permeation of acetic acid and lactic acid was suppressed mainly due to the electrical repulsion at high pH. For caffeine, the effect of molecular size on permeation was larger than that of other species. The permeation of phenol was substantially higher than that of other species because its electrical repulsion was relatively small and it was smaller than caffeine, which probably resulted in lower permeation suppression.

## Figures and Tables

**Table 1:** Properties of compounds used for the permeation test [31].

Compound	Formula	Molecular Weight (g/mol)	pKa
Acetic acid	CH_3_COOH	60.05	4.756
Lactic acid	CH_3_CH(OH)COOH	90.08	3.858
Phenol	(C_6_H_5_)OH	94.11	9.99
Caffeine	C_5_HO_2_N_4_(CH_3_)_3_	194.19	10.4

**Table 2:** Permeances of acetic acid, phenol, and caffeine in mixed aqueous solution.

Species	Permeance (m/s)		
	pH 1.1	pH 2.9	pH 8.1
Acetic acid ^1^	2.16 × 10^−7^	8.82 × 10^−8^	0
Phenol ^1^	4.10 × 10^−7^	3.01 × 10^−7^	2.72 × 10^−7^
Caffeine ^2^	1.41 × 10^−7^	7.94 × 10^−8^	4.19 × 10^−8^

^1^ 0.1 mol/L initial concentration; ^2^ 0.02 mol/L initial concentration.

## Data Availability

The data presented in this study are available on request to the corresponding author.

## Supplementary Materials

The following supporting information can be downloaded at: https://www.mdpi.com/article/10.3390/membranes13010082/s1, Figure S1: SEM image of cross section of membrane obtained each step.
