# Supplementary material for: Preparation and Permeation Properties of a pH-Responsive Polyacrylic Acid Coated Porous Alumina Membrane
Source: Membranes (Basel). 2023 Jan 9;13(1):82. doi: 10.3390/membranes13010082 (PMC9863411; doi:10.3390/membranes13010082)
Supplement: Supplementary file 1 [file membranes-13-00082-s001.zip › membranes-2097403-supplementary.pdf]

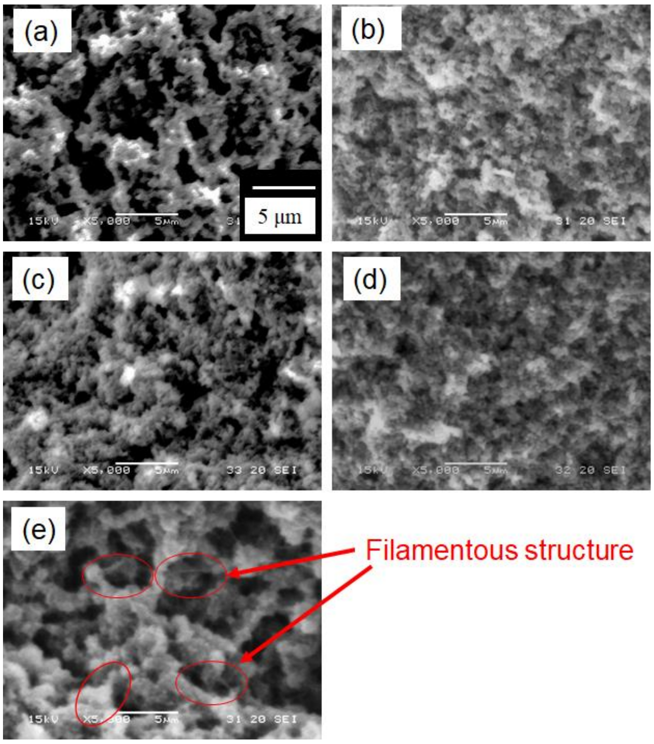

Figure S1. SEM image of cross section of membrane obtained each step (a: Raw support, b: After first step, c: After second step, d: After third step, e: After fourth step with 4 times of polymerization).
